# Clinical Spectrum of SCID: The Key is in the Thymus?

**DOI:** 10.3389/fimmu.2014.00111

**Published:** 2014-03-19

**Authors:** Mirjam van der Burg, Menno C. van Zelm

**Affiliations:** ^1^Department of Immunology, Erasmus MC, University Medical Center, Rotterdam, Netherlands

**Keywords:** severe combined immunodeficiency, Omenn syndrome, thymic epithelial cells, hypomorphic mutation, T-cell, immune deregulation

Genetic defects in the recombination activating genes, RAG1 and RAG2 are known to impair V(D)J recombination in developing B and T-cells, thereby causing T-B-severe combined immunodeficiency (SCID) (1). Importantly, RAG defects with residual recombination activity (hypomorphic mutations) give rise to the Omenn syndrome, which is characterized by erythroderma, eosinophilia, hyper IgE, and the presence of oligoclonal autoreactive T-cells (2). In recent years, additional patients with RAG mutations were identified that presented with atypical clinical features, raising important questions regarding diagnosis, and treatment strategies (3). Furthermore, the underlying mechanism explaining how this clinical heterogeneity can be caused by mutations in these RAG genes remained unclear. Recently, Lee et al. determined the level of residual recombination activity of mutant RAG proteins and found that it correlated well with the clinical phenotype of the patients (4). However, this was only part of the story because IJspeert et al. showed that 22 patients with similar RAG mutations presented with different clinical presentations (5) Thus, the varying degrees of immune dysregulation in RAG-deficient patients cannot be predicted solely based on the RAG gene defect.

The study of Rucci et al. in *Frontiers in Immunology* provided new insight into the role of thymic stroma in immune regulation (6). Following earlier observations of abnormalities in thymic stroma and impaired expression of AIRE and tissue specific antigens (TSA) in thymus of Omenn syndrome patients (7, 8), the authors studied two murine models of leaky SCID: *rag1^S/S^* mice with hypomorphic S723C substitutions in the *rag1* gene and *lig4^R/R^* mice with R278H mutations in the gene encoding DNA ligase IV. The *rag1^S/S^* mice display severe combined immunodeficiency with residual development of oligoclonal and functionally impaired T-cells, and some mice develop features consistent with Omenn syndrome (9). The *lig4^R/R^* mice have a leaky SCID phenotype without features of Omenn syndrome (10).

Both *rag1^S/S^* and *lig4^R/R^* mice showed a significant reduction in thymus size and cellularity due to decreased absolute numbers of CD4^+^CD8^+^ double positive (DP) as well as of CD4 and CD8 single positive (SP) thymocytes. Thymic development was arrested incompletely at DN3 stage, consistent with a V(D)J recombination defect. Newly generated SP thymocytes in both models underwent maturation in the medulla, but this intra-thymic maturation was accelerated as evidenced by skewing toward a more mature phenotype, probably accompanied by homeostatic proliferation in a lymphopenic microenvironment (11). The *rag1^S/S^* and *lig4^R/R^* models differed in the diversity of the thymic TCR repertoire: this was polyclonal in thymocytes of *lig4^R/R^* mice, whereas *rag1^S/S^* mice displayed a highly restricted TCR repertoire, similar to that of Omenn syndrome patients.

To study the nature of this difference, the authors subsequently focused on thymic epithelial cells (TEC). Cortical (c)TECs and medullary (m)TECs play a critical role in thymic selection and central tolerance (Figure 1A), and *cross-talk* with developing thymocytes is crucial for TEC maturation and for maintenance of thymic architecture (12). The thymic demarcation between the cortex and the medulla was preserved *lig4^R/R^* mice, whereas it was completely absent in *rag1^S/S^* mice. In *rag1^S/S^* mice, this disrupted architecture also resulted in the absence of AIRE-expressing mTECs. AIRE regulates the expression of genes encoding peripheral tissue-specific antigens (TSAs). Presentation of TSA peptides by mTECs or dendritic cells leads to tolerance via clonal deletion of self-reactive thymocytes or by facilitating differentiation into natural regulatory T (nTreg) cells. nTreg cells were severely reduced in number, but still displayed a regulatory function. This is in contrast to Omenn syndrome patients that show disturbed nTreg function.

**Figure 1:**
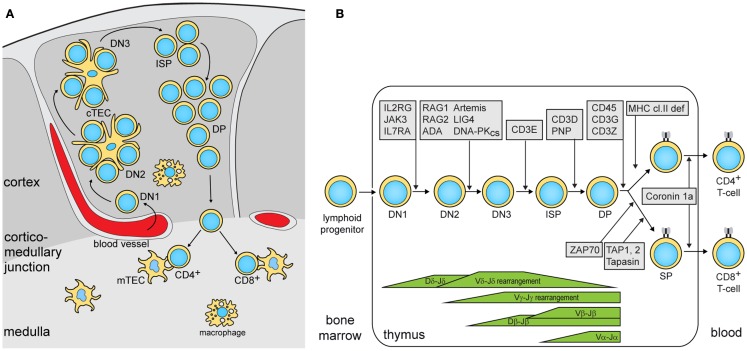
**Precursor T-cell differentiation in human thymus**. **(A)** Thymocyte differentiation and their migration through the anatomic niches. The earliest double negative (DN) thymocytes enter the thymus at the cortico-medullary junction and migrate to the sub-capsular zone. In humans, 3 DN subsets can be identified: DN1, CD34^+^CD38^−^CD1a^−^; DN2, CD34^+^CD38^+^CD1a^−^; DN3, CD34^+^CD38^+^CD1a^+^ (14). Subsequently, these upregulate CD4 and become immature single positive (ISP), followed by CD3^+^CD4^+^CD8^+^ double positive (DP) and finally into CD3^+^CD4^+^ or CD3^+^CD8^+^ single positive (SP) cells while passing through the cortex and the medulla. **(B)** Human T-cell differentiation stages including V(D)J recombination bars (14) and genetic defects underlying PID that result in impaired precursor differentiation. The indicated developmental blocks largely rely on data from targeted mutation studies in the mouse.

In summary, the authors showed that the degree by which hypomorphic mutations impair T-cell development is associated with the defects in thymic stroma architecture and AIRE and TSA expression in mTECs. Especially, when the differentiation block only allows differentiation of a few thymocytes with a restricted TCR gene repertoire, the thymus defects lead to immune dysregulation. Both mouse models showed similar reduction in thymus size and cellularity. Still, *rag1^s/s^* mice showed a restriction in their TCR gene repertoire as compared with *lig4^R/R^*. Thus, despite the differential impact of these mutations on V(D)J recombination, homeostatic processes in the thymus compensated the lower production in *rag1^s/s^* mice with additional proliferation. The association of immune dysregulation with TCR repertoire restriction has recently been confirmed through next generation sequencing of TCRβ gene rearrangements in patients with hypomorphic SCID and immune dysregulation (13). These patients had mutations in RAG1/2, the common γ-chain of the IL-2 receptor and ζ-associated protein kinase 70 (ZAP70), which all affect T-cell differentiation prior to the SP stage (Figure 1B).

Thus, the article by Rucci et al. has begun to provide a rationale for the various degrees of immune dysregulation in patients with Omenn syndrome or atypical SCID. Central tolerance in the thymus appears to play a key role and seems to be influenced by the combination of the generated TCR repertoire and the “TSA repertoire” as expressed by mTECs and DC. The clinical phenotype of hypomorphic mutations will depend on the level of residual activity of the affected protein, the number, the immunophenotype, and clonality of the generated thymocytes, and via these on the thymic architecture and the number and “diversity” of mTECs. The prospects of high-throughput sequencing of TCR gene rearrangements will likely provide further insights into the diversity in immune dysregulation in SCID.

## References

[1] Schwarz K Gauss GH Ludwig L Pannicke U Li Z Lindner D RAG mutations in human B cell-negative SCID. Science (1996) 274:97–9 10.1126/science.274.5284.97 8810255

[2] Villa A Santagata S Bozzi F Giliani S Frattini A Imberti L Partial V(D)J recombination activity leads to Omenn syndrome. Cell (1998) 93:885–96 10.1016/S0092-8674(00)81448-8 9630231

[3] Niehues T Perez-Becker R Schuetz C More than just SCID – the phenotypic range of combined immunodeficiencies associated with mutations in the recombinase activating genes (RAG) 1 and 2. Clin Immunol (2010) 135:183–92 10.1016/j.clim.2010.01.013 20172764

[4] Lee YN Frugoni F Dobbs K Walter JE Giliani S Gennery AR A systematic analysis of recombination activity and genotype-phenotype correlation in human recombination-activating gene 1 deficiency. J Allergy Clin Immunol (2013). 10.1016/j.jaci.2013.10.007 24290284 PMC4005599

[5] IJspeert H Driessen GJ Moorhouse MJ Hartwig NG Wolska-Kusnierz B Kalwak K Similar recombination-activating gene (RAG) mutations result in similar immunobiological effects but in different clinical phenotypes. J Allergy Clin Immunol (2014). 10.1016/j.jaci.2013.11.028 24418478 PMC7112318

[6] Rucci F Poliani PL Caraffi S Paganini T Fontana E Giliani S Abnormalities of thymic stroma may contribute to immune dysregulation in murine models of leaky severe combined immunodeficiency. Front Immunol (2011) 2:15 10.3389/fimmu.2011.00015 21822418 PMC3150116

[7] Cavadini P Vermi W Facchetti F Fontana S Nagafuchi S Mazzolari E AIRE deficiency in thymus of 2 patients with Omenn syndrome. J Clin Invest (2005) 115:728–32 10.1172/JCI23087 15696198 PMC546458

[8] Poliani PL Facchetti F Ravanini M Gennery AR Villa A Roifman CM Early defects in human T-cell development severely affect distribution and maturation of thymic stromal cells: possible implications for the pathophysiology of Omenn syndrome. Blood (2009) 114:105–8 10.1182/blood-2009-03-211029 19414857 PMC2710940

[9] Walter JE Rucci F Patrizi L Recher M Regenass S Paganini T Expansion of immunoglobulin-secreting cells and defects in B cell tolerance in Rag-dependent immunodeficiency. J Exp Med (2010) 207:1541–54 10.1084/jem.20091927 20547827 PMC2901061

[10] Rucci F Notarangelo LD Fazeli A Patrizi L Hickernell T Paganini T Homozygous DNA ligase IV R278H mutation in mice leads to leaky SCID and represents a model for human LIG4 syndrome. Proc Natl Acad Sci U S A (2010) 107:3024–9 10.1073/pnas.0914865107 20133615 PMC2840307

[11] Datta S Sarvetnick N Lymphocyte proliferation in immune-mediated diseases. Trends Immunol (2009) 30:430–8 10.1016/j.it.2009.06.002 19699149

[12] Shores EW Van Ewijk W Singer A Disorganization and restoration of thymic medullary epithelial cells in T cell receptor-negative scid mice: evidence that receptor-bearing lymphocytes influence maturation of the thymic microenvironment. Eur J Immunol (1991) 21:1657–61 10.1002/eji.1830210711 2060577

[13] Yu X Almeida J Darko S van der Burg M Deravin SS Malech H Human syndromes of immunodeficiency and dysregulation are characterized by distinct defects in T-cell receptor repertoire development. J Allergy Clin Immunol (2014). 10.1016/j.jaci.2013.11.018 24406074 PMC3972286

[14] Dik WA Pike-Overzet K Weerkamp F de Ridder D de Haas EF Baert MR New insights on human T cell development by quantitative T cell receptor gene rearrangement studies and gene expression profiling. J Exp Med (2005) 201:1715–23 10.1084/jem.20042524 15928199 PMC2213269

